# Regeneration of mature dermis by transplanted particulate acellular dermal matrix in a rat model of skin defect wound

**DOI:** 10.1007/s10856-012-4745-9

**Published:** 2012-08-19

**Authors:** Haibin Zuo, Daizhi Peng, Bixiang Zheng, Xiaoling Liu, Yong Wang, Lihua Wang, Xin Zhou, Jing Liu

**Affiliations:** 1 Institute of Burn Research, Southwest Hospital, Third Military Medical University, 29 Gaotanyan Street, Chongqing, 400038 China; 2 Tissue Engineering Research Unit, State Key Laboratory of Trauma, Burns and Combined Injury, Third Military Medical University, Chongqing, 400038 China

## Abstract

Native mammalian extracellular matrix (ECM) has been made in various forms including particles, sheet and mesh which are appropriate for site-specific applications. The ECM particles are usually created by homogenization method and have a wider size distribution. This needs to be improved to produce more uniform ECM particles. In present study, we had successfully developed a method for preparing particulate acellular dermal matrix (PADM) in different gauges. The resultant PADM was approaching a rectangular parallelepiped or cubic shape, with a better or narrower size distribution than other ECM particles in previous reports. It also retained ultrastructure and functional molecules of native ECM. In vivo performances were evaluated after implantation of PADM in an acute full-thickness skin defect wound in rats. Histological analysis showed that allogeneic PADM used as dermal regeneration template could facilitate maturation and improving collagen bundle structure of regenerated dermis at the endpoint of 20 weeks post-surgery. The PADM could be used for further investigation in analyzing the impacts of cellularly and/or molecularly modified PADM on soft tissue regeneration.

## Introduction

Mammalian ECM, derived from various tissues and organs, has been used as a biologic scaffold for therapeutic regenerative applications [1, 2]. Removal of cells from a tissue or an organ leaves the structural and functional molecules that constitute the ECM [3, 4]. These molecules in acellular ECM, such as collagen and sulfated glycosaminoglycan (sGAG), facilitate the communication of the adjacent cells with each other and with external environment [5]. The native acellular ECM as a template scaffold for the remodeling of tissues and organs plays a critical role in tissue regeneration.

Acellular ECM can be configured into several different forms including sheet, mesh, particulate, or tube-shaped form which are appropriate for site-specific applications [2]. Human acellular dermal matrix (ADM) in both sheet (AlloDerm) and particulate (Cymetra) forms are increasingly utilized for substituting dermal defect [6, 7], soft tissue augmentation [8–10], dermatology [11], otolaryngology [12], and sinus tracts [13, 14].

Currently, mechanical disruption of ADM material under its wet state was one of methods to produce available spreadable or injectable ECM particles. The uniformity of particle size and shape is dependent upon the source and composition of ECM and upon the method by which the particle is formed [15]. Cymetra is created by homogenizing AlloDerm strips at liquid nitrogen temperature, 68 % of which with diameter ranging from 58 to 593 μm [8]. However, ECM particles with diameter for 52 μm or less are apt to host phagocytosis and do harm to the function of host phagocytes [8]. Furthermore, the geometry of ECM particles is a crucial factor in determining its suitability as a substratum for anchorage-dependent cell adhesion, migration, proliferation and differentiation in vivo [16, 17]. Consequently, the granulating process of ADM needs to be improved to produce more uniform PADM, especially the size and profile of particles.

In present study, we had successfully established a method to prepare PADM in different gauges. The obtained PADM was approaching a rectangular parallelepiped or cubic shape, with a narrower size distribution than other particles in previous reports [8]. It also retained ultrastructure and functional molecules of the ECM. In terms of dermal regeneration application, allogeneic PADM in gauge 0.5 mm was implanted underneath the autologous split-thickness skin graft (STSG, 0.20 mm thick) to heal the acute full-thickness skin defect wound in rats. In vivo performances of implanted PADM on dermis regeneration were evaluated by macroscopic observation and histological analysis of the healed wound at 20 weeks post-surgery.

## Materials and methods

### Source of skin tissue

Male Sprague-Dawley (SD) rats (*N* = 8) weighing 380–420 g were provided by Experimental Animal Center, Institute of Surgical Research, Third Military Medical University, and used as full-thickness skin donors. Donor rats were anaesthetized by intraperitoneal injection of 1 % (w/v) pentobarbital (45 mg/kg) and then sacrificed by exsanguination. After dorsal hair was shaved, the full-thickness skin was excised and scrubbed with 0.1 % benzalkonium bromide solution for 15 min and povidone-iodine solution for 5 min. The subdermal fat tissue, epidermis and partial dermis were removed from completely cleaned skin with sterile phosphate buffered saline (0.01 M PBS, pH 7.4) by using a dermatome. The obtained reticular dermal matrix (DM) with a thickness of 0.20 mm was washed three times in PBS and transversely bisected, when one part was assigned for ADM preparation as described below and the other for control DM samples. These samples were wrapped in PBS-moistened gauze, sealed in sterile plastic bag, and stored at −20 °C until acellular process or dry.

### Preparation of ADM

The ADM was prepared by the modified method [18]. Briefly, the frozen donor reticular DM samples (*N* = 8) were thawed and soaked in double distilled water for 30 min at room temperature (22–24 °C). Then, they were treated with 0.25 % (w/v) trypsin (Amresco, USA)/EDTA–Na_2_ at 37 °C for 2 h with continuous shaking, and were thoroughly rinsed in PBS at 22–24 °C for three times (10 min each). Subsequently, the DM samples were incubated in 0.5 % (v/v) Triton X-100 (Amresco, USA) for 24 h at 22–24 °C with continuous shaking to remove cellular components from dermal matrix. Finally, the samples were thoroughly rinsed in PBS at 22–24 °C for three times (10 min each). The obtained rat reticular ADM samples (*N* = 8) were then placed in sterile plastic bags and stored at −20 °C until vacuum-dry. All solutions were filter sterilized and all procedures were performed aseptically.

### Histological and biochemical assessment of DM and ADM

#### Histological observation

The cell and DNA in the reticular ADM and DM samples were assessed by hematoxylin and eosin (H&E) and 4′-6-diamidino-2-phenylindole (DAPI) staining, respectively. Samples of DM and ADM were fixed in 10 % buffered neutral formaldehyde and embedded in paraffin. A series of sections were analyzed with H&E staining and then observed under light microscope (IX71, Olympus, Japan). Tissue samples stored at −20 °C were also prepared for DAPI staining by frozen sections. After embedded in OCT compound, frozen samples were cut into sections, washed with distilled water and 30 % isopropanol to remove the OCT compound, and then stained with DAPI to evaluate presence of visible nuclear material. The sections were observed under light microscope (DM 6000B, Leica, Germany).

#### Papain digestion

Dried DM (*N* = 8) and ADM (*N* = 8) tissues (~5 mg) were digested with 1 mg/ml papain (Worthington Biochemical, USA) solution in buffer (50 mM Na_2_HPO_4_, 38 mM EDTA–Na_2_, 35 mM l-cysteine) at 65 °C for 24 h. The samples were centrifuged (10,000 g, 2 min, 4 °C) and the supernatants were stored at 4 °C for further analyses.

#### Quantification of DNA

A DNA quantitation kit (Sigma, USA) for 96-well plate, fluorescence assay was used to quantify the DNA content in the papain-digested supernatant. According to manufacturer’s instructions, a Bisbenzimide H 33258 solution for 0.1 μg/ml was prepared in 10× fluorescence assay buffer. DNA standard solutions were added into Bisbenzimide H 33258 solution to achieve final concentrations from 50 to 500 ng/ml for a standard curve of calf thymus DNA. 180 μl of Bisbenzimide H 33258 solution was added into each well with 20 μl of the papain-digested DM or ADM supernatants. Fluorescence intensity of DNA standards, DM and ADM supernatants at 360 nm excitation and 460 nm emission were measured using microplate reader (Varioskan flash, Thermo, USA). The DNA concentration in DM and ADM samples was calculated from the standard curve and expressed as μg of DNA per mg of dry weight of tissue.

#### Quantification of sGAG

A dimethylmethylene blue (DMMB) (Sigma, USA) spectrophotometric assay was used to quantify the level of sGAG content in papain-digested DM or ADM supernatants [19]. The DMMB assays were performed in 96-well plates. 20 μl of standards containing up to 50 μg/ml bovine chondroitin sulfate B (Sigma, USA) and the papain-digested DM or ADM supernatants were individually added to 200 μl of DMMB stock solution and optical density (OD) was immediately measured at 490 nm by microplate reader (Varioskan flash, Thermo, USA). The sGAG concentration in DM and ADM samples was calculated from the standard curve, and expressed as μg of chondroitin sulfate per mg of dry weight of tissue.

### Production of PADM

Before vacuum-dry, DM and ADM sheets were removed from −20 °C freezer and immersed in double distilled water at room temperature to thaw. The DM and ADM sheets were dried for 8 h (10^−1^ mbar) in a freeze dryer (Savant 2081, Thermo, USA), and then chopped into particles in different gauges (such as 0.2, 0.5, 0.7, 1.2 mm) at room temperature. The processed PADM was sterilized by soaking in 75 % ethanol and stored at 4 °C for future use.

### In vitro assessment of PADM’s characteristics

#### Mean diameters and size distribution

The particle mean diameters and size distribution of processed PADM in four gauges (including 0.2, 0.5, 0.7, 1.2 mm) were determined by laser diffraction (MS2000, Malvern, UK) [20]. Briefly, 800 ml of the distilled water was added into a glass beaker with 1,000 ml capacity, and then the glass beaker was transferred to the particle size analyzer (Hydro 2000mu, Malvern, UK) under moderate stirring (manufacturer’s pump speed 3,000 r/min setting). The size distribution of particles was defined as a function of particle diffraction using Mastersizer 2000 software and values plotted as a percent of amount. In order to evaluate size distribution of these four gauges PADM, their percentile undersize diameters 10 % (d_0.1_), 50 % (d_0.5_) and 90 % (d_0.9_) were calculated.

#### Morphological analysis

The morphological profiles of PADM (gauge: 0.5 mm) were observed under light microscope and scanning electron microscopy (SEM). The paraffin-embedded PADM sections were stained with H&E solution for light microscopical analysis. A thin layer of rehydrated PADM was mounted on a slide connecting to adhesive metallic tape. The samples were observed under SEM (S-3400, Hitachi, Japan) in ESED model.

For transmission electron microscopy (TEM) observation, rehydrated PADM (gauge: 0.5 mm) samples were immersed in 2.5 % glutaraldehyde. After primary fixation, PADM samples were post-fixed in 1 % osmium tetroxide, and dehydrated in a graded series of acetone, after which they were embedded in the resin. Thin sections were cut and mounted on mesh copper grids. After stained in uranyl acetate and lead citrate, TEM (Tecnait-10, Philips, Netherlands) was used to investigate particles’ ultrastructure.

#### Fourier transform infrared spectroscopy (FTIR) examination

FTIR spectra of dried particulate DM (PDM) (gauge: 0.2 mm) and PADM (gauge: 0.2 mm) were recorded on a spectrum (GX, PerkinElmer, USA) using KBr disk method [21]. They were individually mixed with ~5 times of vacuum dried KBr and pressed into pellets by hydraulic press. The spectra were obtained at the resolution of 2 cm^−1^ in region of 4000–400 cm^−1^.

### PADM as dermal regeneration template in skin wound healing

#### Wound model and implantation procedure

Twelve male SD rats as recipients weighing 220–250 g were divided into experimental (E) group (*N* = 6) and control (C) group (*N* = 6) according to random number table. An acute full-thickness skin defect with original area of 4 × 6 cm was produced on the dorsum of each anaesthetized rat. Autologous thin STSG (0.20 mm thick) was made from the excised full-thickness skin (4 × 6 cm area) of the same rat using a dermatome. The prepared allogeneic PADM (gauge: 0.5 mm) at expansion rate of 10:5 was evenly implanted on the dorsal wound and then covered with autologous STSG of 4 × 6 cm area in E group. The used allogeneic PADM (gauge: 0.5 mm) in E group was prepared from vacuum-dried ADM sheet of donor rats. The ADM sheet with 4 × 6 cm area was weighed and recorded, and then chopped into particles in 0.5 mm gauge at the room temperature. Half of the weight of the particles from the 4 × 6 cm ADM sheet was used in each rat in E group. According to the paper coating method of area measurement [22, 23], it is assumed that the area ratio of two pieces of the same substance with uniform thickness is equal to the weight ratio of them. Consequently, the expansion rate of PADM was 10:5. Skin defects in C group were only covered with autologous STSG of 4 × 6 cm area without PADM underneath. The non-meshed STSG in both E and C groups was sutured between the STSG and the edge of skin defect wound discontinuously by silk suture, and was then covered by vaseline gauze and povidone-iodine gauze. The wound dressings were changed at 1 week post-surgery. Dorsal wounds of anaesthetized rats were sterilized by povidone-iodine solution. This study was performed according to the guidelines in the *Guide for the Care and Use of Laboratory Animals* published by the US National Institutes of Health (NIH).

#### Macroscopic observation of wound healing

To determine the survival rate, contraction rate of transplanted skin, the recipient rats were anaesthetized by intraperitoneal injection of 1 % (w/v) pentobarbital (45 mg/kg) to shave the dorsal hairs around the wound at post-surgery weeks (PSW) 4, 8, 12 and 20. All wounds were imaged by digital camera (FE-360, Olympus, Japan) immediately, and at PSW 4, 8, 12 and 20 respectively. All photographs were taken with experimental rats placed adjacent to a metric ruler that was used for area calibration, allowing subsequent planimetric quantitative analysis by the *Image J* software (Version 1.38×, NIH, USA).

Percent graft survival = (A_X−_A_N_)/A_X_ × 100 %, where A_N_ is necrotic graft area on week X post-surgery, and A_X_ is total wound area on week X post-surgery.

Percent graft contraction or expansion = (A_0_−A_X_)/A_0_ × 100 %, where A_0_ is wound skin area immediately after surgery, and A_X_ is total wound area on week X post-surgery.

Notes: positive value represents contraction, negative value represents expansion.

#### Histological analysis

The tissues of healed wounds and adjacent normal skin were harvested for assessment of collagen’s structure and arrangement in regenerated dermis in both groups at PSW 20. These biopsies (~0.5 × 1.5 cm) were fixed in 10 % buffered neutral formaldehyde and embedded in paraffin for histological analysis. Tissue sections were stained with H&E, or with sirius red (direct red 80) (Sigma-Aldrich, USA) in picric acid and photographed under cross-polarized light under light microscope (IX71, Olympus, Japan), respectively, to evaluate the dermal regeneration in healed skin [24].

Histologic review of the biopsies was blinded performed. Planimetric quantitative analyses, such as the mean diameter of collagen bundles(μm), gap rate between collagen bundles(%), type I collagen content(%), type III collagen content(%), and the ratio of type I to type III collagen content were measured by the *Image*-*Pro Plus (IPP)* software (Version 6.00, Media Cybernetics, USA).

### Statistical analysis

All data were expressed as mean ± SD. Survival rate and contraction or expansion rate of both groups at different PSW were tested by an independent-samples *t* test, Levene test, and *t’* test. All the indexes of collagen, such as the mean diameter of collagen bundles(μm), gap rate between these bundles(%), type I collagen content(%), type III collagen content(%), and the ratio of type I to type III collagen of both groups and surrounding normal skin also were tested by an independent-samples *t* test, Levene test, and *t’* test. All analyses were performed using SPSS software(Version 13.0, SPSS, USA) for Windows. *P* < 0.05 was considered statistically significant.

## Results

### Histological and biochemical assessment of DM and ADM

**Fig. 1:**
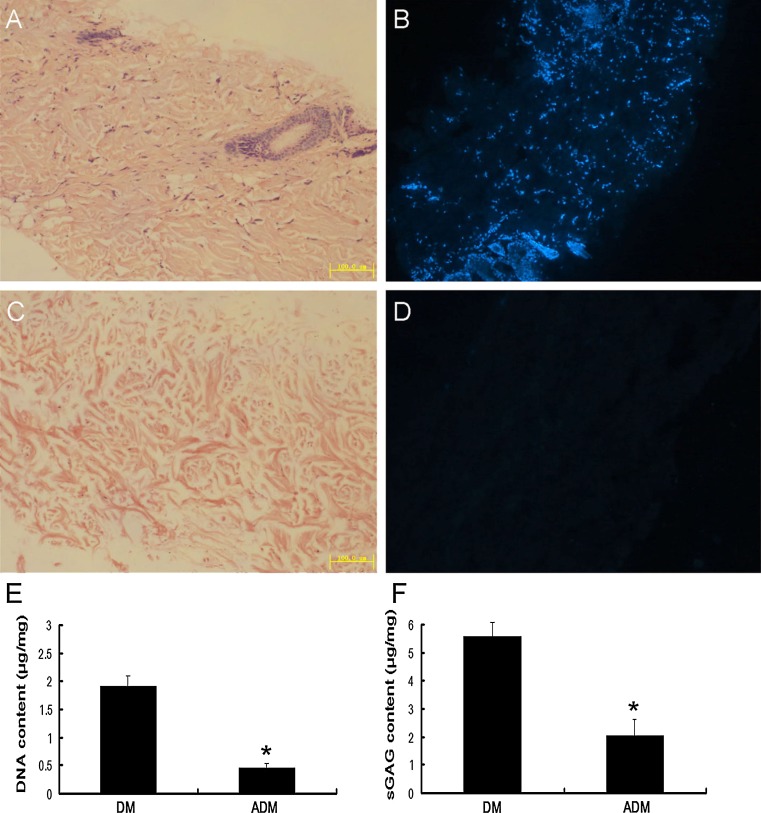
Histological and biochemical assessment of DM and ADM. **a**, **b** H&E and DAPI stainings of reticular DM (×100). (**c**, **d**) H&E and DAPI stainings of reticular ADM (×100). **e**, **f** DNA and sGAG content in reticular DM and ADM. *P < 0.05, as compared with DM

As shown in Fig. 1, before decellularization, H&E and DAPI stainings of the reticular DM sections showed massive nucleus in ECM, positive staining was mainly localized in the hair follicle of reticular dermis (Fig. 1a, b). After decellularization, ADM sections showed few positive staining of residual nuclear material by H&E (Fig. 1c) and DAPI (Fig. 1d) stainings. Bisbenzimide H 33258 fluorescence assay also showed a significant decrease of DNA content from 1.91 ± 0.18 μg/mg dry weight in DM to 0.46 ± 0.08 μg/mg dry weight in ADM (Fig. 1e). Compared with DM samples, sGAG content in ADM was decreased from 5.58 ± 0.47 to 2.02 ± 0.60 μg/mg (Fig. 1f).

### In vitro characteristics of PADM

**Fig. 2:**
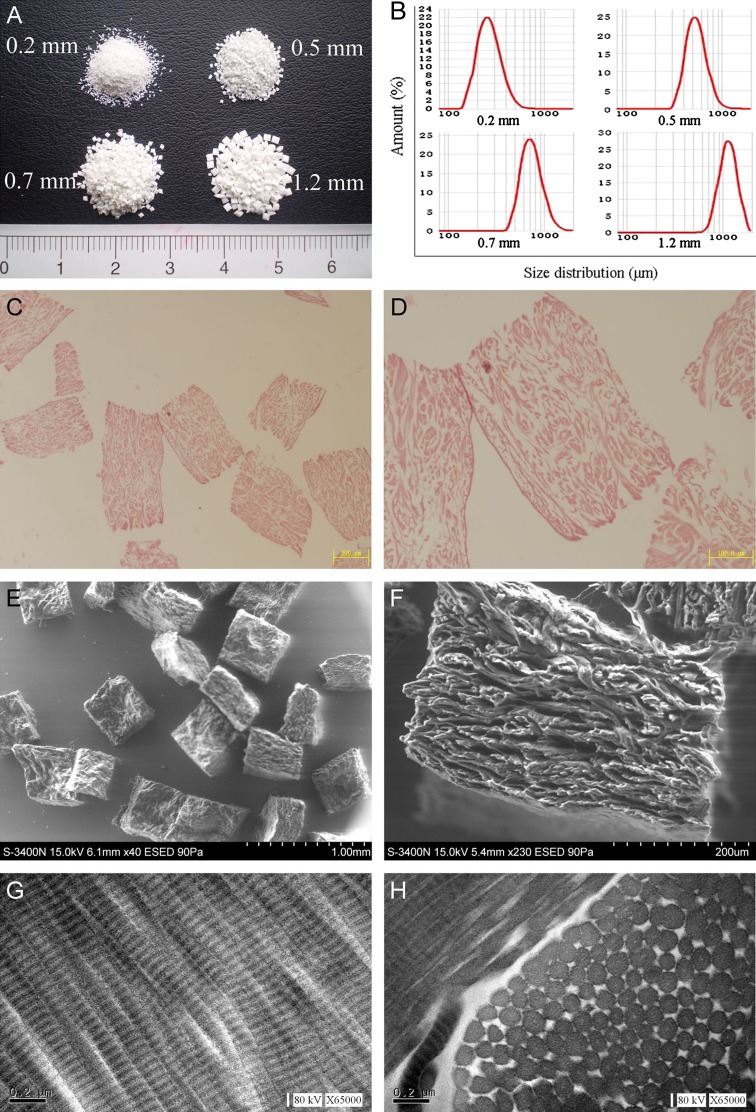
Macroscopic, microscopic and ultrastructural features of PADM. **a** PADM powders of four gauges. **b** Size distribution of these PADM in four gauges (0.2, 0.5, 0.7, 1.2 mm). **c**, **d** H&E staining of PADM in 0.5 mm gauge. **e**, **f** SEM micrographs of PADM in 0.5 mm gauge. **g**, **h** TEM micrographs of PADM in 0.5 mm gauge

The PADM samples were white powders (Fig. 2a). The D [3, 2] (surface-weighted mean diameters) and D [4, 3] (volume-weighted mean diameters) of PADM in four gauges were shown in Table 1. Size distribution of PADM in four gauges showed 80 % (from d_0.1_ to d_0.9_) with diameters for 194–380 μm (gauge: 0.2 mm), 400–737 μm (gauge: 0.5 mm), 530–1027 μm (gauge: 0.7 mm) and 856–1485 μm (gauge: 1.2 mm), respectively (Table 1; Fig. 2b). The median particle sizes (d_0.5_) of PADM in four gauges were also shown in Table 1.

**Table 1:** Mean diameters and size distribution of PADM in different gauges

Gauges (mm)	Mean diameters (μm)		Size distribution		
	D [3, 2]	D [4, 3]	d_0.1_ (μm)	d_0.5_ (μm)	d_0.9_ (μm)
0.2	323	350	194	263	380
0.5	625	668	400	529	737
0.7	875	948	530	715	1,027
1.2	1,261	1,311	856	1,136	1,485

D [3, 2] is the surface weighted mean diameter; D [4, 3] is the volume weighted mean diameter; d_x_ is the percentile undersize diameter

The collagen bundles of PADM was shown in Fig. 2c, d, which presented in the photomicrographs of H&E stained PADM (gauge: 0.5 mm) sections. Furthermore, its geometry was approximately a rectangular parallelepiped shape (Fig. 2e). There were fibrous collagen bundles on the cross-section of PADM in SEM micrograph (Fig. 2f). By TEM, a sequential arrangement of cyclical transverse striation was shown in Fig. 2g. Regularity of fibrillar spacing and uniformity in fibril size were presented in Fig. 2h.

**Fig. 3:**
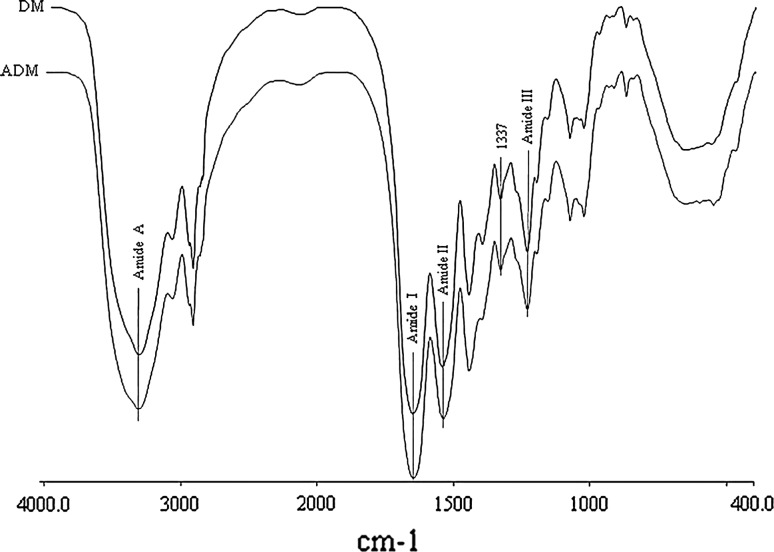
FTIR spectra of the PDM and PADM in gauge of 0.2 mm. The amide A, amide I, amide II and amide III peaks around 3329, 1659, 1549 and 1239 cm^−1^ are characteristic of the NH stretching, α-helix, β-sheet, and β-turn, respectively. The 1,337 cm^−1^ peak in both samples is characteristic of type I collagen

FTIR spectra of PADM and native PDM were shown in Fig. 3. Compared with native PDM, PADM protein backbone was reserved as confirmed by the presence of characteristic amide A, amide I, amide II and amide III peaks at 3329, 1659, 1549 and 1239 cm^−1^, respectively [25]. The 1,337 cm^−1^ peak in both samples was one of a number of peaks in range of 1,400–1,260 cm^−1^ which were characteristic of type I collagen [26].

### Macroscopic observation of wound healing

**Fig. 4:**
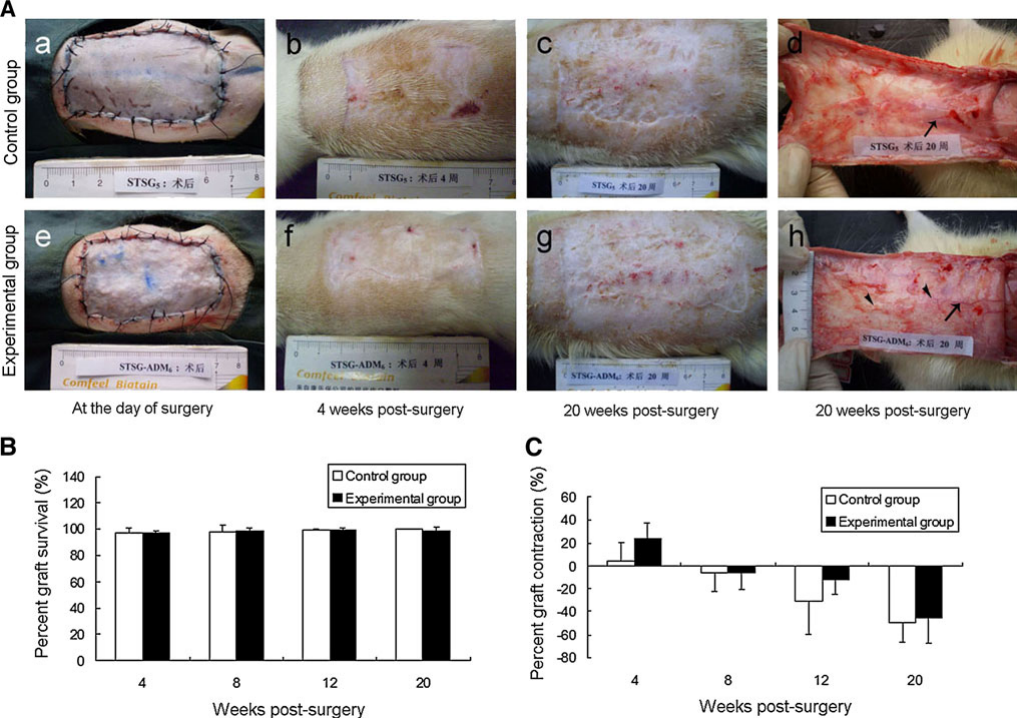
Macroscopic observation of healed wound in control and experimental groups. **a** Macroscopic observation of healed wound in C group (**a**, **b**, **c**) and E group (**e**, **f**, **g**) immediately, and at PSW 4, 20. (*Ad* and *Ah*) Macroscopic observation of subdermal tissue side of healed wound from C group and E group at PSW 20, respectively. **b** The percent graft survival of both groups at PSW 4, 8, 12 and 20. **c** The percent graft contraction of both groups at PSW 4, 8, 12 and 20

Macroscopic observation of wounds in C group and E group immediately, and at PSW 4, and 20 were shown in Fig. 4a. Percent graft survival and percent graft contraction or expansion of both groups manifested no statistical difference at PSW 4, 8, 12 and 20 (Figs. 4b, c).

### Histological analysis of wound healed skin

**Fig. 5:**
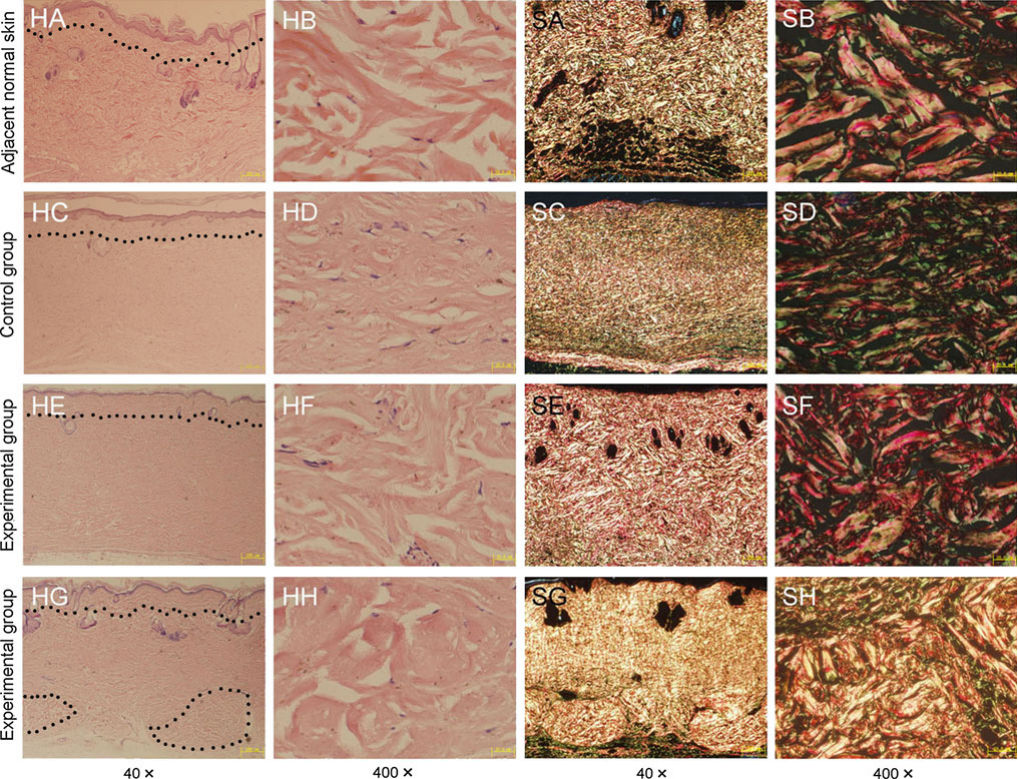
Histological analysis of healed wound in control and experimental groups. (*HA*–*HH*) H&E staining photomicrographs of adjacent normal skin (*HA*, *HB*) and healed wound sections from C group (*HC*, *HD*) and E group (*HE*–*HH*). (*SA*–*SH*) *Sirius red* staining photomicrographs of adjacent normal skin (*SA*, *SB*) and healed wound sections from C group (*SC*, *SD*) and E group (*SE*–*SH*). *Dot line* indicated the 0.2 mm depth of control adjacent skin and transplanted STSG. *Dot circle* in Fig. 5 HG indicated the residual PADMs

H&E staining photomicrographs of adjacent normal skin and healed wound sections from both groups were shown in Fig. 5HA–HH. Collagen bundles in C group were positioned in slightly parallel and more compact arrays, which showed homogenization of collagen. Collagen bundles in E group (Fig. 5HE, HF) were arranged in dendritic arrays, which was similar to the pattern in normal skin (Fig. 5HA, HB). Furthermore, in E group, H&E staining analysis showed few fibroblasts migration into the incomplete degraded PADM at PSW 20 (Fig. 5HG). The incompletely degraded PADM in recipient healed dermal tissue was also shown in hyaline degeneration with different sizes of scattered bubbles (Fig. 5HH). The E group had much thicker collagen bundles [(9.61 ± 0.82) μm vs. (7.26 ± 1.39) μm, (*P* < 0.01)] and had a significant increase in the gap rate between these collagen bundles [(23.82 ± 5.00) % vs. (16.94 ± 4.17) %, (*P* < 0.05)] than that of C group. Mean diameters of collagen bundles and gap rates between these collagen bundles in both groups and adjacent normal skin by HE staining were shown in Table 2.

**Table 2:** Mean diameters of collagen bundles and gap rates between these collagen bundles in both groups and adjacent normal skin by HE staining

Groups	Mean diameters of collagen bundles (μm)	Gap rate between collagen bundles (%)
Normal skin	13.81 ± 0.95	36.90 ± 5.27
Control	7.26 ± 1.39^a^	16.94 ± 4.17^a^
Experimental	9.61 ± 0.82^a,b^	23.82 ± 5.00^a,b^

^a^ *P* < 0.05, as compared with normal skin

^b^ *P* < 0.05, as compared with control group

Sirius red staining photomicrographs of adjacent normal skin and healed wound from both groups were shown in Fig. 5SA–SH. Under polarized light, type I collagen was thick fibres with strongly birefringent yellow or red, and type III collagen was thin fibres with greenish and weakly birefringent. And the existing PADM were appearing strongly birefringent yellow/red under polarized light (Fig. 5SG, SH). About more than 60 % amount of implanted PADM had been degraded and integrated into recipient healed dermal tissue. Compared with C group, E group had significant increase in type I collagen content [(80.21 ± 5.39) % vs. (68.05 ± 8.41) %, (*P* < 0.05] and the ratio of type I to type III collagen content [(4.31 ± 1.15) vs. (2.33 ± 0.98), (*P* < 0.01)]. Above mentioned indexes of collagen in wound of E group were similar to these of surrounding peri-wound normal skin, which were shown in Table 3.

**Table 3:** Contents of type I or III collagen and the ratio of type I to type III collagen in both groups and adjacent normal skin by sirius red staining

Groups	Type I collagen content (%)	Type III collagen content (%)	Ratio of type I to type III collagen
Normal skin	83.54 ± 2.28	16.46 ± 2.28	5.19 ± 0.91
Control	68.05 ± 8.41^a^	31.95 ± 8.41^a^	2.33 ± 0.98^a^
Experimental	80.21 ± 5.39^b^	19.79 ± 5.39^b^	4.31 ± 1.15^b^

^a^ *P* < 0.05, as compared with normal skin

^b^ *P* < 0.05, as compared with control group

## Discussion

Several sheet dermal substitutes [27], such as AlloDerm, XenoDerm, Integra and Pelnac have been reported that they can facilitate skin wound healing in combination with autologous STSG. When wound beds were covered by the sheet dermal substitutes, the inflammatory exudates or/and blood silting may accumulate underneath them [28, 29]. Obviously, PADM has better drainage effect than sheet dermal substitutes and would reduce the complications of hydrops and hematoma. Although the sheet dermal equivalent could be meshed at the expansion rate from 1.5:1 to 6:1 and used in the treatment of massive third degree burns [30], the mesh holes are often bigger than the gaps between PADMs. Furthermore, the distances between PADMs could be more exactly controlled by accounting the used amount of PADM by its weight. Therefore, the PADM could also avoid the formation of schistic or netty scar which is derived from using meshed sheet dermal equivalent. Since 1985, autologous microskin grafting has been widely used in wound repair for massive deep burn wound [31]. The resultant healed wound skin is often scarring and less flexible due to its lack of dermis. PADM could be more conveniently implanted in combination with autologous microskin grafting, and provide a potential approach for the improvement of wound healing quality in major burn patients.

The uniformity of particle size and shape is dependent upon the source and composition of the ECM and the method by which the particle is formed [15]. Currently, Cymetra was created by cutting AlloDerm sheets into strips and homogenizing strips at liquid nitrogen temperature [32]. The time and duration of cryogenic fractionation step often depended upon the homogenizer utilized. However, Cymetra showed shapes that like broken bits, and 68 % of Cymetra had their diameters ranging from 58 to 593 μm [8, 33]. In our study, dried ADM sheets were chopped into strips with desired widths or gauges, which could be selected from 100 to 2,000 μm. The obtained vacuum-dried ADM strips with desired widths were then chopped into particles with corresponding gauges at room temperature. The geometry of prepared PADM had a more well-arranged shape than that of Cymetra. All the ratios of d_0.9_ to d_0.1_ in these four gauges PADM were less than or equal to twofold, which indicated that all the sizes of these PADM were well distributed.

The modified decellularization method in our study had effectively removed nucleus and adnexal components of rat dermis [34], and more than one-third of amount of sGAG content was reserved in the ADM. Glycosaminoglycans in ECM have special impact on delaying wound contraction and inducing regeneration [35]. Another research in our lab found that ~40–50 % of the initial amounts of TGF-β1, bFGF and KGF in the ADM were retained after decellularizating process (our unpublished data). SEM observation was shown that this PADM had a loose structure at the cross sections. The retained growth factors and sGAG as well as the loose structure of PADM would facilitate the migration and proliferation of fibroblasts and vascular endothelial cells, and eventually promote the revascularization and dermal regeneration of implanted PADM.

Watari et al. [17] found that the production of superoxide anions, cytokines TNF-α and IL-1β from neutrophils are increased with the decrease of particle size, especially below 10 μm. Recently, He et al. [36] also found that the control of particle size and surface charge of nanocarriers would affect its cellular uptake and distribution in vivo. Furthermore, Sampath et al. [16] reported that fine matrix with particle size of 44–74 μm did not induce bone in vivo. The inability of fine matrix to induce bone is explained by the crucial role of matrix geometry in triggering the biochemical cascade of endochondral bone differentiation in vivo. Severely burned patients suffer an early hyperinflammatory response and late immunosuppression [37, 38]. The PADM with uniform size and diameter for >52 μm will have a potential application in regenerating the dermis and do not aggravate the existed immune dysfunction in massive deep burn patients.

Microscopic and ultrastructural features of the matrix play important roles in facilitating migration of the selected cell types into ECM scaffold [27]. By TEM, collagen fibrils in rat reticular DM were more closely packed, as compared with that in human reticular DM [our unpublished data], which resembled the results of rat and human bladder tissue reported by Dahms et al. [28]. FTIR analysis was performed aiming to characterize the impact of decellularization on collagen fibril’s native molecular structure stabilization of the PADM. The α-helix, β-sheet, and β-turn conformations of polypeptide chain gave rise to distinct and distinguishable positions of the amide I, II, and III peaks [39, 40]. The peak at 1,337 cm^−1^ in collagen was attributable predominantly to the so-called wagging vibration of proline side chains [26]. There was no significant peak shift with decellularization process in preparing ADM from rats. The FTIR results demonstrated that the PADM retained the collagen fibril’s native molecular structure.

In mammals, skin normally responds to injury by the processes of inflammation, epithelialization, formation of granulation tissue, wound contraction, extracellular-matrix reorganization and scar formation [41]. In the scenarios of spontaneous wound healing without skin grafting, however, loose-skinned mammals (such as rodents) may experience complete wound closure and little scarring due to contraction [42, 43]. If a unique acellular dermal regeneration template or moderate thickness of STSG is placed into the wound, wound contraction is strongly inhibited, and the skin undergoes a regenerative-type healing [3, 44–46]. Therefore, dermal regeneration template or STSG with moderate dermis play an important role in the process of skin wound healing.

At the initial stage of PADM and STSG being implanted on wound, the plasma nutrients and oxygen for STSG survival are derived from wound basement. Compared with the use of sheet dermal substitutes, PADM with better effects of drainage and penetration could provide more nutrition and oxygen to the covered STSG. So, PADM could be implanted combined with autologous STSG by one-stage surgery, avoid two-stage surgery. The sheet dermal equivalent usually takes 2–3 weeks for sufficient vascular ingrowth to overlay the skin graft which will be almost fully survival [47]. The prepared PADM was implanted in combination with autologous STSG by one-stage surgery in this study for preclinical testing of a therapeutic dermal regeneration.

In this study, the original area (4 × 6 cm) of the rat wound model was more clinically relevant than small area wound models, such as areas of 1 × 1 cm or 2 × 2 cm that was often used in mice or rats. The graft survival rates and contraction rate of both groups were not significantly different with or without PADM implantation at different PSW. The reason is that the skin structure of rat has a marked difference from human epidermis structure. Rat epidermis contains only 2–3 layers of cells, but human epidermis has 7–14 layers of cells. So, the prepared autologous STSG of 0.2 mm from rats remains a certain amount of dermis tissue and might act as dermal regeneration template. Consequently, very low contraction of healed wound was observed in both control and experimental groups.

At the endpoint of study, both groups had an expansion of the transplanted STSG. This is mainly due to the increased body weight and body surface area as rat grows up. At PSW 20, the weights of rats in both groups were increased from 220–250 to 520–620 g. In accordance with the increase of body surface area over time, the areas of healed wound with transplanted STSG in both E and C groups were enlarged. So, the positive values of percent graft contractions in both groups were decreased. When the areas of healed wound skins were larger than those of the initial wounds immediately after surgery, the negative values of percent graft contractions represent percent graft expansion. Although the expansion of transplanted STSG in E group seemed to be different from that in C group, there was not statistically significant between them. For this topic, we could make further research to optimize the amount and gauge of transplanted PADM for a better effect of wound healing.

Sclafani et al. [9] research found that the wholly degradation time of implanted AlloDerm was more than 6 months. Therefore, 20 weeks after implantation was selected as the endpoint of this study to observe the wholly degradation time of implanted PADM in vivo. The PADM as natural dermal substitute mainly consists of three types of macromolecules [48]. They are collagen fibres, proteoglycans (PGs), and biological active factors. Although these three types of macromolecules play critical roles in the wound healing, the fate of PADM during skin regeneration is mostly determined by the fate of predominant collagen fibers. For collagen fibres, we could observe that the incompletely degraded PADM in recipient healed dermal tissue was shown in hyaline degeneration with different sizes of scattered bubbles. Most of the implanted PADM had been degraded and integrated into the healed dermal tissue of recipients at PSW 20. There are merely several approaches to track the degradation of implanted PAMD in vivo. Gilbert et al. [49] described a ^14^C labeling quantitative method for evaluating the degradation of biologic scaffold materials. For tracing collagen fibres of PAMD by its shape and HE staining in this study, we observed that the incompletely degraded PADM in recipient healed dermal tissue was shown in hyaline degeneration with different sizes of scattered bubbles. Approximately 40 % of the initial amounts of implanted PADM were retained at PSW 20.

The presence of type III collagen is prominent in the initial stage of wound healing and scar-tissue formation [50]. As fetal development proceeds and as healing tissue gains in strength, type III collagen is replaced by the stronger type I collagen [48]. So, the ratio of type I to type III collagen content in regeneration dermis could be served as the index for judging the maturity of regeneration dermis. The STSG in combination with PADM had a similar content and alignment of type I collagen fibres and type III collagen fibres in regenerated dermis in skin defect wound healing to normal skin. There were less type III fibres in the experimental group and normal skin than that in control group. These data indicated that the implantation of PADM could facilitate the maturity of regenerated dermis and potentially reduce wound scarring. In our study, a traditional approach with qualitative images like sirius red staining were used for relative quantitative analyses. Talwar et al. [51] assessed the relation between type I and type III procollagen precursor levels by radioimmunoassay, western blot, and immunohistology in punch biopsy specimens from human skin. These techniques, such as western blotting and real time (RT)-PCR, could be performed to substantiate some of these findings at the levels of both protein and mRNA expressions of type I and type III collagen in our further research.

The obtained PADM could be potentially used in the regeneration of tissue or organs by the methods of spreading, injecting, spraying or packing. The particles may also be combined with stem cells or growth factors to enhance the acceptance and regenerative capability of implanted particulate biomaterial. Further research is needed to investigate the size and profile effect of ECM particles on various tissues regeneration and their correspondent mechanism of matrix geometry in triggering the biochemical cascade of the regeneration tissue differentiation in vivo.
